# A quality improvement initiative to increase HIV post-exposure prophylaxis treatment for emergency department patients after sexual assault: A pre-post study

**DOI:** 10.1371/journal.pone.0320690

**Published:** 2025-03-28

**Authors:** Douglas A.E. White, Montana Jewett, Molly Burns, Cedric Rodriguez, Cinthya Mujica Pinto, Gabriela Regalado, Kevin Del Angel, Hillary J. Larkin, Erik S. Anderson

**Affiliations:** Department of Emergency Medicine, Alameda Health System, Oakland, California, United States of America; Tarbiat Modares University, IRAN, ISLAMIC REPUBLIC OF

## Abstract

**Background:**

Many emergency department (ED) patients after a sexual assault face barriers to receiving HIV post-exposure prophylaxis (PEP). We implemented a quality improvement initiative which updated the sexual assault electronic health record (EHR) template and made available free, full-course PEP treatment packs for use at provider discretion. The aim of this study was to compare the receipt of HIV PEP for ED patients receiving sexual assault care before and after the initiative.

**Methods:**

This was a retrospective, quasi-experimental, pre-post study of all ED patients who completed a sexual assault examination between June 1, 2022 – January 31, 2023 (pre-intervention) and March 1, 2023 – October 31, 2023 (post-intervention). An odds ratio (OR) and 95% confidence interval (CI) were calculated to determine the initiative’s effect on PEP prescribing. Multivariable logistic regression models estimated the independent effect of the initiative while controlling for potential confounders.

**Results:**

Of the 235 sexual assault examinations, 117 (49.8%) were during the pre-intervention and 118 (50.2%) were during the post-intervention periods. Pre-intervention, the mean age was 33.0 years (standard deviation [SD] 12.3), 94.0% were female, 30.8% Black, 31.6% Latinx, and 28.2% White. Post-intervention, the mean age was 29.2 years (SD 12.6), 90.7% were female, 42.4% Black, 28.8% Latinx, and 17.8% White. Patients were more likely to receive HIV PEP post-intervention (33/118, 28.0%) than pre-intervention (11/117, 9.4%) (OR 3.7, 95% CI 1.8 to 7.8). The independent effect of the initiative on HIV PEP prescribing remained significant after controlling for demographics (OR 3.6, 95% CI 1.6 to 8.0), assault characteristics (OR 4.2, 95% CI 1.6 to 11.1), and provider experience (OR 3.5, 95% CI 1.7 to 7.5).

**Conclusion:**

The availability of HIV PEP treatment packs plus a modification to the EHR template led to a significant increase in the provision of HIV PEP for ED patients after sexual assault.

## Background

United States (US) emergency departments (ED) provide care to tens of thousands of patients after sexual assault each year [1–3]. This care includes providing trauma-informed medical and forensic examinations, screening for sexually transmitted infections (STI), and initiating post-exposure prophylaxis (PEP) for STIs, including the initiation of HIV PEP [4–6].

HIV PEP, the use of antiretroviral medications after a single high-risk event (such as may occur with sexual assault), is highly effective in preventing HIV seroconversion if taken as soon as possible after the exposure (within 72 hours) and with strict adherence to a 28-day regimen [7].

The risk of HIV transmission after sexual assault is not known but felt to be low, with the per-act risk of HIV seroconversion extrapolated from consensual vaginal (0.08%) and receptive rectal intercourse (1.38%) [7,8]. Because of this, the Centers for Disease Control and Prevention recommend HIV PEP to be considered on a case-by-case basis and after an assessment of assault-related factors that might increase the risk of HIV exposure and transmission. These factors include the likelihood that the assailant has HIV (is a man who has sex with other men or uses injection drugs) or exposure characteristics (did rectal or vaginal penetration occur or were mucosal injuries sustained) [7,9–11].

For many patients in whom HIV PEP is indicated after a sexual assault, receiving PEP in a timely manner and accessing a full treatment course remains a significant barrier [12]. A national survey of nearly 600 US EDs showed that only 65.3% of EDs offered HIV testing and HIV PEP to patients receiving care after a sexual assault [13]. Even when EDs routinely provide HIV testing and HIV PEP after sexual assault, initial compliance with PEP depends on the ability of a patient to first obtain the prescription, which can be impeded by limited access to pharmacies and cost. And for patients who just survived sexual assault, making a separate trip to an outpatient pharmacy may be emotionally triggering and further hinder the ability to obtain medications [12].

To circumvent these barriers, the standard practice for ED HIV PEP delivery in the US is to provide a first dose of an FDA-approved regimen while the patient is still in the ED followed by a prescription for the remaining 27 days [14]. To facilitate short term compliance, some EDs will provide patients with a 3-5 day bridging starter pack in conjunction with a standard prescription [15–17]. Providing the entire 28-day PEP medication supply at the initial visit is another strategy to increase the likelihood of adherence, especially when patients face difficulties returning for follow-up visits [18]. A recent study by Cherabie *et al* demonstrated an innovative model where 87% of patients at risk for HIV acquisition after sexual assault were provided a full 28-day, meds-in-hand, supply of HIV PEP [19].

In February of 2023, at the Alameda Health System (AHS), Highland Hospital ED, we implemented a quality improvement initiative, “sexual assault PEP” (SA PEP), to increase the receipt of HIV PEP for sexual assault patients. The initiative included two interventions: 1) a modification to the sexual assault electronic health record (EHR) template to include a field prompting examiners to discuss PEP and 2) making available free 28-day supplies of meds-in-hand HIV PEP therapy (PEP treatment packs) for use at the discretion of the sexual assault examiners.

The purpose of this study was to compare the receipt of HIV PEP for ED patients receiving sexual assault care in the period before (pre-intervention) and after the SA PEP initiative (post-intervention).

## Methods

### Study design and subjects

This was a retrospective, quasi-experimental pre-post study of a quality improvement initiative. We included all ED patients who completed a sexual assault examination between June 1, 2022 – January 31, 2023 (pre-intervention period) and March 1, 2023 – October 31, 2023 (post-intervention period). We chose this time period based on a consensus agreement among authors that 8 months was a reasonable duration of time to observe both an effect and sustainability of the intervention. The initiative was rolled out in February 2023, which served as a one-month washout period. Patients receiving a sexual assault examination during February 2023 were therefore excluded. We also excluded patients for whom PEP was not indicated (either because they were living with HIV infection or presented ≥72 hours after the assault) or who were transferred from an outside facility and received a first dose of PEP as well as a 28-day prescription by the transferring hospital.

The AHS Institutional Review Board approved the study with a waiver of written informed consent.

### Setting

This study was performed in the ED at Highland Hospital in Oakland, California, a level-1 trauma center with an annual census of 57,000 visits. The ED supports a well-established routine HIV screening program and provides rapid antiretroviral therapy (ART) using Biktarvy® (bictegravir, emtricitabine, tenofovir alafenamide) starter packs (donated by Gilead, Sciences) to eligible patients [20]. The Highland Hospital ED serves as the county referral center for sexual assault forensic examinations.

At the Highland Hospital ED, sexual assault forensic examinations are conducted by the department’s pool of 20 advanced practice providers (APP) who have specialized training in conducting forensic examinations, with support from a sexual assault patient advocate. Forensic assault examinations are performed within 5 days of the assault, after approval by the responsible law enforcement agency, and with the consent of the patient. The Highland ED performs approximately 180 sexual assault examinations each year, accounting for 75% of the sexual assault examinations in Alameda County for patients ≥ 14 years of age. Using a structured template, a written log with basic patient information and characteristics of the assault is completed by the APP and maintained in a locked cabinet in the sexual assault examiners’ office.

### Pre-intervention sexual assault HIV PEP workflow

Prior to the SA PEP initiative, the protocol for ED HIV PEP was Truvada® (emtricitabine and tenofovir disoproxil fumarate) plus Tivicay® (dolutegravir) or Isentress® (raltegravir) for eligible patients presenting within 72 hours after sexual assault [21]. Eligibility for HIV PEP was based on the clinical judgment of the treating APP and was not strictly mandated. By protocol, patients were given the first doses of HIV PEP (available in the ED pyxis) as soon as possible during the index visit and then prescribed a complete course of treatment upon discharge (28 days total). Additionally, APPs could use an EHR “sexual assault” template to facilitate accurate and comprehensive documentation of encounters for sexual assault.

### Sexual assault HIV PEP intervention

In January of 2023, an internal review of pharmacy records identified low numbers of ED HIV PEP prescriptions and potential missed opportunities for PEP among patients evaluated after sexual assault. This prompted a meeting between the ED Director of HIV Screening (DAEW) and the APPs to discuss barriers to prescribing PEP. Citing concerns that prescriptions often go unfilled because of pharmacy copays, lack of insurance coverage, and patient difficulties accessing outpatient pharmacies for PEP, the APPs requested approval to use the Biktarvy® starter packs for HIV PEP after sexual assault. They also requested improving the sexual assault EHR template to include a field “reminding” them to assess HIV PEP eligibility.

The decision to expand the indications of Biktarvy® to PEP for sexual assault patients was approved from the Division Chief of HIV Medicine, the HIV clinical pharmacy lead, and backed by recommendations of the East Bay Getting to Zero collaborative that included Biktarvy® as one of three preferred HIV PEP regimens [22]. A modification to the sexual assault template included the addition of embedded fields reminding providers to 1) discuss the risks and benefits of PEP and 2) document whether PEP was given.

On February 15, 2023, the APPs were notified at a departmental staff meeting and by email that Biktarvy® starter packs could be used for HIV PEP for sexual assault patients at their discretion. APPs were instructed to obtain baseline laboratory testing to ensure the safe use of Biktarvy®, which included having liver function tests < 5 times the upper limit of normal, an estimated glomerular filtration rate > 30 mL/min, and a negative pregnancy test. APPs were instructed to provide eligible patients with 4 starter packs, equaling a 28-day course of PEP (each starter pack consisted of a labeled, sealed bottle containing 7 tablets). This was referred to as an HIV PEP treatment pack. If desired, standard PEP prescriptions using any FDA-recommended regimen could be provided instead of the Biktarvy® treatment packs. Eligibility for HIV PEP remained unchanged, based on the clinical judgment of the treating APP, and was not strictly mandated.

### Outcome measure

The primary outcome measure was the number and proportion of patients undergoing sexual assault examinations who were provided PEP in the pre-intervention compared with the post-intervention period.

### Data abstraction

The sexual assault database was used by trained research staff to identify all sexual assault forensic examinations completed between June 1, 2022, through October 31, 2023. Using a structured data collection instrument, patient level data for each forensic examination were abstracted from a review of the EHR (Epic Systems). The instrument had both closed response and free text fields. Data included patient demographics (age, gender, race, ethnicity), discharge time, treating APP, assault characteristics (type of penetration, condom use), whether PEP was offered and provided, and details of any PEP prescription or treatment pack given, including medication type and duration. Data was accessed for research purposes on 11/9/2023, 11/20/2023, 11/29/2023, 3/21/2024, and 3/22/2024. The authors had access to information that could identify individual participants during and after data collection. Study data were collected and managed using REDCap electronic data capture tools hosted at the AHS [23,24]. REDCap is a secure web application for building and managing online surveys and databases.

### Definitions

Receipt of PEP was defined as EHR documentation that an HIV PEP prescription was electronically signed by the treating provider or an HIV PEP treatment pack was given. Patients that received only a first dose of HIV PEP while in the ED, but no additional prescription or treatment pack, were not considered as being provided HIV PEP.

Incorrect PEP treatment (PEP prescribing errors) was defined as either an incorrect PEP regimen (inadequate treatment, such as Truvada® only) or an incorrect duration of treatment (total duration fewer than 28 days). Correct PEP treatment was defined as a 28-day prescription of an approved HIV PEP regimen.

Risk of HIV acquisition from the sexual assault was defined as high (condomless receptive rectal or vaginal sex), moderate (condomless insertive rectal or vaginal sex), and low (insertive or receptive oral sex, exposure to blood, saliva, urine, sex with a condom) according to Pacific AIDS Education and Training Center Criteria [22].

APPs were categorized as high, moderate, or low volume examiners based on the number of sexual assault examinations performed during the study period. Categories were determined posteriori and based on tertiles of completed sexual assault examinations.

### Statistical analysis

Descriptive statistics were computed for all variables. Continuous variables were reported as medians with interquartile ranges (IQR) and means with standard deviations (SD) and categorical variables as proportions with 95% confidence intervals (CI), where appropriate.

An odds ratio (OR) and 95% CI were calculated to determine the effect of the SA PEP initiative on PEP prescribing for sexual assault patients. Posteriori, three multivariable logistic regression models were used to estimate the independent effect of the SA PEP initiative while controlling for potential confounders. Model 1 controlled for patient characteristics (age, gender, race/ethnicity), model 2 controlled for assault characteristics (assailant known, risk of HIV risk exposure, discharge time), and model 3 controlled for number of sexual assault examinations performed by APPs. Statistical analyses were performed on de-identified data with Stata (version 14; StataCorp).

## Results

**Fig 1:**
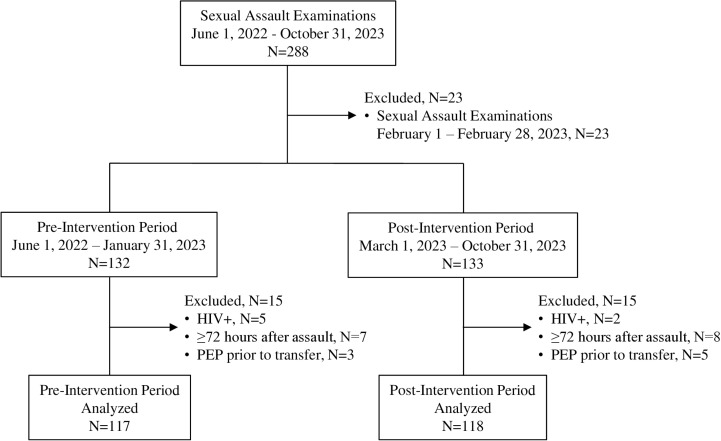
Consort Diagram Study Selection Process.

Between June 1, 2022, and October 31, 2023, 279 patients aged ≥ 14 years underwent 288 sexual assault examinations. Of these, 53 encounters were excluded from the analysis because they occurred during the month of February 2023 (*N* =  23), were patients living with HIV (*N* =  7), received PEP at a different facility prior to transfer (*N* =  8), or presented ≥ 72 hours after the assault (*N* =  15), leaving 235 sexual assault encounters in the study population. Of the 235 sexual assault examinations, 117 (49.8%) were during the pre-intervention period and 118 (50.2%) were during the post-intervention period (Fig 1).

Overall, the mean patient age was 31.1 years (SD + /-12.5 years); 217 (92.3%) were female; 86 (36.6%) were Black, 54 (23.0%) were White, and 71 (30.2%) were Latinx. One hundred nineteen (50.6%) assaults were by a known assailant and 127 (54.0%) were documented as being at a high risk for HIV exposure. One hundred and eleven (61.3%) of the sexual assault examinations were completed by high-volume APP examiners (≥15 examinations). Only 31.1% of sexual assault patients were discharged during daytime hours (7am-3pm). Although patients in the pre-intervention period were slightly older than those in the post-intervention period(mean age 33.0 years, SD + /-12.3 years vs. mean age 29.2 years, SD + /- 2.6 years, P = 0.02), there did not appear to be any clinically meaningful differences in characteristics between the study periods (Table 1).

**Table 1:** Characteristics of Patients, Assailants, and Advanced Practice Providers for Emergency Department Sexual Assault Examinations Before and After Sexual Assault PEP Initiative.

	Total *N* = 235	Pre-Intervention Period *N* = 117	Post-Intervention Period N = 118	*P*-value
Age, mean (SD), years	31.1 (12.5)	33.0 (12.3)	29.2 (12.6)	0.02
Gender, N (%)				0.99
Male	14 (6.0)	6 (5.1)	8 (6.8)	
Female	217 (92.3)	110 (94.0)	107 (90.7)	
Non-binary/3^rd^ gender/Other	4 (1.7)	1 (0.9)	3 (2.5)	
Race/Ethnicity, N (%)				0.54
Black	86 (36.6)	36 (30.8)	50 (42.4)	
Latinx	71 (30.2)	37 (31.6)	34 (28.8)	
Asian	12 (5.1)	4 (3.4)	8 (6.8)	
White	54 (23.0)	33 (28.2)	21 (17.8)	
Native Hawaiian or Other Pacific Islander	0	0	0	
American Indian or Alaska Native	2 (0.9)	1 (0.9)	1 (0.9)	
Other	4 (1.7)	2 (1.7)	2 (1.7)	
Unknown	6 (2.6)	4 (3.4)	2 (1.7)	
Exchange sex for money/nonmonetary items	18 (7.7)	5 (4.3)	13 (11.1)	0.05
Language, N (%)				0.38
English	202 (89.8)	102 (87.2)	100 (84.8)	
Spanish	21 (9.3)	11 (9.4)	10 (8.5)	
Other	2 (0.9)	4 (3.4)	8 (6.8)	
Assailant				0.97
Known to patient	119 (50.6)	58 (49.6)	61 (51.7)	
Not Known to patient	73 (31.1)	39 (33.3)	34 (28.8)	
Unknown	43 (18.3)	20 (17.1)	23 (19.5)	
Time from assault to exam				0.95
≤ 72 hours	215 (91.5)	107 (91.5)	108 (91.5)	
Unknown	20 (8.5)	10 (8.5)	10 (8.5)	
HIV risk from assault^*^				0.95
High	127 (54.0)	61 (52.1)	66 (55.9)	
Moderate	1 (0.4)	1 (0.8)	0	
Low	25 (10.6)	13 (11.1)	12 (10.2)	
Unknown	82 (34.9)	42 (35.9)	40 (33.9)	
Sexual assault examinations completed^**^				
High volume examiner	111 (61.3)	72 (61.5)	72 (61.0)	0.45
Moderate volume examiner	72 (30.6)	39 (33.3)	33 (28.0)	
Low volume examiner	19 (8.1)	6 (5.1)	13 (11.0)	
Time of ED discharge				
7am-3pm	73 (31.1)	38 (32.5)	35 (29.7)	0.64

Abbreviations: PEP, post-exposure prophylaxis; SD, standard deviation; N, number.

Wilcoxon rank sum test or Fisher’s exact test was used to compute P-values for continuous or categorical data, respectively. For comparisons between three or more groups, one-way ANOVA was used.

* HIV risk from the sexual assault was defined as high (condomless receptive rectal sex and condomless receptive vaginal sex), moderate (condomless insertive rectal sex or condomless insertive vaginal sex), and low (insertive or receptive oral sex, exposure to blood, saliva, urine, sex with a condom) [22].

** There were 20 APPs who performed sexual assault examinations. APPs were categorized as high volume (≥15 examinations each), moderate volume (7-14 examinations each), or low volume (≤6 examinations each) examiners.

Use of the sexual assault template was similar between the study periods (pre-intervention: 98/117, 83.8%; post-intervention: 101/118, 85.6%, P = 0.70). Documentation of offering HIV PEP was infrequently recorded in the pre-intervention period (14/117, 12.0%) compared to the post-intervention period(91/118, 77.1%) (P < 0.0001).

During the pre-intervention period, 11 of the 117 patients completing sexual assault examinations (9.4%) were provided PEP compared to 33 of the 118 patients (28.0%) during the post-intervention period (OR 3.7, 95% CI 1.8 to 7.8) (Table 2). Of the 33 patients receiving PEP during the post-intervention period, 22 (66.7%) received treatment packs and 11 (33.3%) received a prescription. Prescribing errors occurred with 3 (13.6%) of the 22 PEP prescriptions (incorrect PEP regimen *N* =  1, incorrect treatment duration *N* =  2) and with none of the 22 PEP treatment packs.

**Table 2:** Receipt of HIV Post-Exposure Prophylaxis for Emergency Department Patients Completing Sexual Assault Examinations Before and After the Sexual Assault PEP Initiative.

	Total *N* = 235	Pre-Intervention Period *N* = 117	Post-Intervention Period *N* = 118	Odds Ratio (95% CI)
Receipt of PEP^*^, N (%)	44 (18.7)	11 (9.4)	33 (28.0)	3.7 (1.8 to 7.8)
Prescription, N (% of PEP received)	22 (50)	11 (100)	11 (33.3)	--
Treatment Pack, N (% of PEP received)	22 (50)	0	22 (66.7)	--

Abbreviations: PEP, post-exposure prophylaxis; CI, confidence interval; N, number.

* Receipt of PEP was defined as EHR documentation that an HIV PEP prescription was electronically signed by the treating provider or an HIV PEP treatment pack was given. Patients that received only a first dose of HIV PEP while in the ED, but no additional prescription or treatment pack, were not considered as being provided HIV PEP.

The results of the three models of multivariable logistic regression are shown in Tables 3–5. After controlling for demographics (model 1: age, gender, and race/ethnicity) (Table 3), assault characteristics (model 2: assailant known status, HIV risk, and time of ED discharge) (Table 4), and APP experience (model 3: high, moderate, low volume examiner) (Table 5), the independent effect of the SA PEP initiative on PEP prescribing remained significant (model 1: OR 3.6, 95% CI 1.6 to 8.0; model 2: OR 4.2, 95% CI 1.6 to 11.1; model 3: OR 3.5, 95% CI 1.7 to 7.5).

**Table 3:** Independent Effect of the Sexual Assault PEP Initiative on the Provision of PEP, Controlling for Patient Characteristics.

Variable	Odds Ratio (95% CI)	Adjusted Odds Ratio (95% CI)
SA PEP Initiative	3.7 (1.8 to 7.8)	3.6 (1.6 to 8.0)
Age, years		
14-23	Ref	Ref
24-34	1.2 (0.6 to 2.7)	1.5 (0.6 to 3.4)
35-44	0.4 (0.1 to 1.4)	0.7 (0.2 to 2.3)
45-54	0.9 (0.2 to 3.6)	1.5 (0.3 to 6.3)
>55	1.2 (0.4 to 4.2)	1.5 (0.4 to 5.9)
Gender Female	0.5 (0.2 to 1.5)	0.5 (0.1 to 2.0)
Race/Ethnicity		
Black	Ref	Ref
White	0.7 (0.3 to 1.7)	0.9 (0.3 to 2.3)
Latinx	0.9 (0.4 to 1.9)	1.1 (0.5 to 2.5)
Other^*^	0.2 (0.1 to 1.7)	0.2 (0.1 to 1.6)

Abbreviations: PEP, post-exposure prophylaxis; SA, sexual assault; CI, confidence interval; Ref, reference.

* Other includes Asian, Native Hawaiian and Pacific Islander, American Indian and Alaskan Native.

**Table 4:** Independent Effect of the Sexual Assault PEP Initiative on the Provision of PEP, Controlling for Assault Characteristics.

Variable	Odds Ratio (95% CI)	Adjusted Odds Ratio (95% CI)
SA PEP Initiative	3.7 (1.8 to 7.8)	4.2 (1.6 to 11.1)
HIV risk from assault Moderate/High^*^^,^^**^	3.5 (0.8 to 15.8)	7.8 (0.9 to 65.7)
Assailant known to patient	0.6 (0.3 to 1.2)	0.3 (0.1 to 0.9)
ED time discharge 7am-3pm	0.8 (0.4 to 1.7)	1.4 (0.5 to 3.7)

Abbreviations: PEP, post-exposure prophylaxis; SA, sexual assault; CI, confidence interval.

* Missing data *N* = 102.

** HIV risk from sexual assault was defined as high (condomless receptive rectal sex and condomless receptive vaginal sex), moderate (condomless insertive rectal sex or condomless insertive vaginal sex), and low (insertive or receptive oral sex, exposure to blood, saliva, urine, sex with a condom) [22].

**Table 5:** Independent Effect of the Sexual Assault PEP Initiative on the Provision of HIV PEP, Controlling for Advanced Practice Provider Experience.

Variable	Odds Ratio (95% CI)	Adjusted Odds Ratio (95% CI)
SA PEP Initiative	3.7 (1.8 to 7.8)	3.5 (1.7 to 7.5)
Sexual assault examinations completed^*^		
High volume examiner	Ref	Ref
Moderate volume examiner	0.2 (0.1 to 0.8)	0.6 (0.3 to 1.4)
Low volume examiner	0.4 (0.1 to 1.1)	2.0 (0.7 to 5.9)

Abbreviations: PEP, post-exposure prophylaxis; SA, sexual assault; CI, confidence interval; Ref, reference.

* There were 20 APPs who performed sexual assault examinations. APPs were categorized as high volume (≥15 examinations), moderate volume (7-14 examinations), or low volume (≤6 examinations) examiners.

## Discussion

EDs play an essential role in the evaluation and care of patients after sexual assault, including providing HIV PEP when assault-related factors are felt to put the patient at increased risk for HIV exposure and transmission [19]. Receiving PEP in a timely manner and accessing a full treatment course are critical for PEP to be effective in preventing HIV transmission after sexual assault and policies to minimize barriers to receiving a full 28-day course of PEP treatment should be prioritized [5–7,12].

This study evaluates the SA PEP program, a quality improvement initiative designed to increase the receipt of HIV PEP for ED patients after sexual assault through two strategies: 1) a modification to the sexual assault EHR template reminding providers to consider and offer PEP and 2) making available free, 28-day HIV PEP treatment packs. Combined, these strategies increased the receipt of HIV PEP three-fold, from 9.4% to 28.0%, for ED patients after sexual assault. Although the increase in PEP prescriptions during the post-intervention period cannot be attributed with 100% certainty to the SA PEP initiative alone, the differences remained statistically significant across our three logistic regression models which controlled for patient demographics, characteristics specific to the assault, and the experience of the sexual assault examiners. Although unmeasured variables may have influenced the provision of PEP in our study, we are not aware of significant changes in APP staffing, APP training, or ED census that occurred between the pre-intervention and post-intervention periods.

Despite minimizing barriers to accessing PEP treatment with the SA PEP initiative, the proportion of patients receiving HIV PEP after sexual assault was relatively low (28%), especially when compared to other published reports of ED HIV PEP after sexual assault [15,16,19,25,26]. A large academic ED in the Northeastern US participating in a sexual assault nurse examiner program reported 124 (69%) of 179 patients receiving sexual assault examinations received HIV PEP over a 3-year period [15]. And a study conducted at the Boston Medical Center ED reported that 82 (45%) of 181 patients evaluated after sexual assault received HIV PEP in the form of a 3-day starter pack [16]. Another study in a large Midwestern ED reported that over a 13-year period, 87% of 423 patients evaluated after a sexual assault where a high-risk HIV exposure was identified received a full course of HIV PEP prior to discharge [19]. The magnitude of differences in HIV PEP treatment rates between these studies and ours may be explained, in part, by specific study exclusion criteria. In our analysis, we included patients regardless of the risk for HIV transmission (post-intervention period excluded only 15 of 133 encounters) whereas in the study by Cherabie *et al*, over 50% of their initial study population of 908 patients with a visit for sexual assault/abuse were excluded, including patients for whom PEP was not offered [19].

We believe the main driver of our low overall PEP numbers may be due to a low acceptance rather than a lack of offering. During the post-intervention period, EHR documentation revealed that the majority of patients (77%) were offered HIV PEP but only 28% accepted PEP, lower than other ED studies which report higher proportions of HIV PEP acceptance after sexual assault, ranging from 32% to 87% [15,16,19,25,26]. The retrospective nature of the study and available data, however, do not allow for an accurate understanding of why PEP acceptance was so low. A better understanding of the true offer and acceptance rate and reasons behind a patient’s decision to accept or decline PEP requires prospective evaluation.

Importantly, we have seen an upward trend in PEP provision and the use of treatment packs with ongoing implementation of our SA PEP initiative, suggesting that the sustainability of this initiative is robust. An analysis of more recent 3 months (February 2024 to April 2024) demonstrate that PEP was provided to 50% of patients completing sexual assault examinations (26/51), with the majority (85%) being treatment packs. Sharing the outcomes from this initiative and eliciting feedback from the APPs at our site will hopefully identify foci for improvement and inform future trainings, educational sessions, and protocols designed to streamline eligibility assessments and increase the acceptance rate of PEP.

We believe that providing HIV PEP medications-in-hand for ED patients after a sexual assault examination is the best strategy to ensure timely and appropriate treatment. Same day treatment for ED patients has been shown to improve compliance and decrease return visits and hospitalizations, especially for those who are underinsured or who are discharged at times when pharmacies are closed [27,28]. Prior studies have demonstrated that providing PEP medications-in-hand for ED patients is feasible and increases the receipt of PEP compared to providing standard prescriptions [29,30]. Compliance with PEP, although not assessed in our study, is further improved when single dose versus multiple dose regimens are used [31,32]. Additionally, we recommend supplying the full 28-day treatment at the time of discharge rather than a short bridging course to an outpatient prescription. Full treatment packs are associated with higher acceptance and completion rates than other PEP treatment strategies and assure that the correct medication regimen and duration are provided [33]. Incorrect PEP prescriptions by emergency physicians are not uncommon [34]. In our study, prescribing errors occurred in 3 (13.6%) of the 22 PEP prescriptions written by physicians and in none of the PEP treatment packs.

How EDs should best go about providing HIV PEP medications to patients after sexual assault, however, is complicated, and policies guiding implementation will need to be developed at the institutional level, with input from multiple departments, balancing cost, pharmacy considerations, compliance, and safety. Providing medications-in-hand, pre-discharge is not standard ED practice. Routinely providing either starter packs or the entire 28-day course requires that EDs stock PEP drugs or have an established agreement with a pharmacy to stock, package, and rapidly dispense PEP drugs.

At our institution, we leveraged an existing hospital Pharmacy and Therapeutics Committee exemption which allowed the use of industry-provided Biktarvy® starter packs for use in the ED, expanding the indications to include HIV PEP. The use of industry-provided starter packs, donated free-of-charge, is often prohibited due to institutional policies and regulatory barriers, which may limit replication of our model.

The use of ED or hospital-based pharmacies, however, can be used to ensure timely access to HIV PEP therapy for ED patients [30,35]. EDs with discharge pharmacies have been shown to reduce barriers like cost, transportation, and pharmacy access when compared to prescriptions requiring filling at local, outpatient pharmacies [35]. Further, many EDs have access to 24-hour inpatient pharmacies and protocols have been developed whereby these pharmacies store, package, and urgently dispense HIV PEP medications to patients prior to discharge [30]. An additional benefit of utilizing existing pharmacy infrastructure is that patients without insurance or with high copayments can be identified immediately and be enrolled in a patient-assistance program or a no-charge compassionate care provision can be applied [7,36].

### Limitations

This was a retrospective evaluation of a single center and the magnitude of influence of our intervention may not be generalizable to other EDs, particularly those with different patient populations, standardized PEP guidelines for sexual assault patients, and improved access to PEP medication dispensing, such as an in-house, 24-hour, 7-days-per-week pharmacy.

The pre-post study design also makes it impossible to attribute the increased provision of PEP solely to the intervention (PEP treatment packs and updated sexual assault template), as other unmeasured factors, such as patient characteristics, examiner practice patterns, or other external events, may influence the primary outcome [37]. Additionally, the relative contribution of the EHR template modification versus the availability of PEP treatment packs with regards to driving the primary outcome measure of PEP prescribing is not known, as these were considered a bundled intervention. Both interventions likely influenced PEP prescribing.

Missing data was not uncommon for several of the clinical variables abstracted from chart review (estimation of risk of HIV acquisition missing 32.8% and knowledge of assailant missing 18.5% of the time) and which were included in logistic regression, possibly reducing the model’s ability to accurately predict dependent variables.

Patient centered outcomes, such as adherence with PEP, follow-up for repeat HIV testing and surveillance for HIV seroconversion, and opportunities to transition patients with ongoing risk for acquiring HIV infection to pre-exposure prophylaxis, were not assessed. Multiple studies have shown that both adherence with HIV PEP and outpatient follow up after ED initiation are extremely low [15,18,19,38,39]. Prospective studies evaluating barriers to adherence and follow-up are needed.

## Conclusion

EDs play an important role in the evaluation and care for patients after sexual assault. A clinical intervention consisting of an EHR template modification plus HIV PEP treatment packs led to a significant increase in the provision of PEP for ED patients evaluated after sexual assault and eliminated errors in treatment. Future research should evaluate medication receipt, adherence, and cost effectiveness of PEP treatment packs compared to prescriptions.
